# Coinfections and Phenotypic Antimicrobial Resistance in *Actinobacillus pleuropneumoniae* Strains Isolated From Diseased Swine in North Western Germany—Temporal Patterns in Samples From Routine Laboratory Practice From 2006 to 2020

**DOI:** 10.3389/fvets.2021.802570

**Published:** 2022-01-28

**Authors:** Isabel Hennig-Pauka, Maria Hartmann, Jörg Merkel, Lothar Kreienbrock

**Affiliations:** ^1^Field Station for Epidemiology, University of Veterinary Medicine Hannover, Bakum, Germany; ^2^Department of Biometry, Epidemiology and Information Processing, WHO Collaborating Centre for Research and Training for Health at the Human-Animal-Environment Interface, University of Veterinary Medicine Hannover, Hannover, Germany; ^3^Department of Infectious Diseases, Institute for Microbiology, University of Veterinary Medicine Hannover, Hannover, Germany

**Keywords:** antibiotic resistance, APP serotype, respiratory disease, influenza virus, porcine reproductive and respiratory syndrome virus, streptococcus suis, susceptibility

## Abstract

*Actinobacillus pleuropneumoniae* (*APP*) is one major bacterial porcine respiratory tract pathogen causing disease outbreaks worldwide, although effective commercial vaccines are available. Due to frequent failure of this preventive measure, treatment with antimicrobials is indispensable to prevent animal losses within an outbreak situation. To preserve the effectivity of antimicrobial substances to fight *APP* should therefore be the primary aim of any interventions. In this study, the temporal development of antimicrobial resistance in *APP* was analyzed retrospectively in the time period 2006–2020 from a routine diagnostic database. In parallel, frequent coinfections were evaluated to identify most important biotic cofactors as important triggers for disease outbreaks in endemically infected herds. The proportion of *APP* serotype 2 decreased over time but was isolated most often from diseased swine (57% in 2020). In ~1% of the cases, *APP* was isolated from body sites outside the respiratory tract as brain and joints. The lowest frequencies of resistant isolates were found for cephalothin and ceftiofur (0.18%), florfenicol (0.24%), tilmicosin (2.4%), tiamulin (2.4%), enrofloxacin (2.7%), and spectinomycin (3.6%), while the highest frequencies of resistant isolates were found for gentamicin (30.9%), penicillin (51.5%), and tetracycline (78.2%). For enrofloxacin, tiamulin, tilmicosin, and tetracycline, significantly lower frequencies of resistant isolates were found in the time period 2015–2020 compared to 2006–2014, while gentamicin-resistant isolates increased. In summary, there is only a low risk of treatment failure due to resistant isolates. In maximum, up to six coinfecting pathogens were identified in pigs positive for *APP*. Most often pigs were coinfected with Porcine Circovirus 2 (56%), *Streptococcus suis* (24.8%), or the Porcine Reproductive and Respiratory Syndrome Virus (23.3%). Potential synergistic effects between these pathogens published from experimental findings can be hypothesized by these field data as well. To prevent *APP* disease outbreaks in endemically infected herds more efficiently in the future, next to environmental trigger factors, preventive measures must also address the coinfecting agents.

## Introduction

*Actinobacillus* (*A*.) *pleuropneumoniae* (*APP*) is causing peracute, acute, and chronic infections in swine worldwide. Surviving pigs which had recovered from disease are persistently infected and harbor *APP* in lung sequesters or on their tonsils (1). In most swine farms with a conventional health status, also clinically healthy pigs are tonsillar carriers of this pathogen. Frequently occurring subclinical diseases in these farms are reflected by recent results of slaughterhouse lung evaluation for dorso-caudal pleurisy suspicious of *APP*. This so-called APP Index calculated per farm was highest in North Western Germany (0.6) which was explained by high pig density and frequent pig transports in this area (2, 3). Due to the high proportion of positive farms, sudden outbreaks of acute disease are in the majority of cases not only linked to the presence of the pathogen but triggered by additional factors as environmental stressors or coinfecting agents. Severe disease outbreaks with high mortality after introduction of the pathogen by carrier animals in a naïve herd are rare but economically devastating events. In both cases, measures to deal with disease are (a) immediate antibiotic treatment to prevent further losses, (b) vaccination, (c) treatment and prevention of coinfecting agents, and (d) identification and elimination of environmental stressors. As prerequisite for choice of an effective antimicrobial for immediate treatment, knowledge about the development of antibacterial resistance in *APP* must be updated from time to time. In addition, the most important coinfecting agents as disease triggers should be known to interpret ambiguous diagnostic findings commonly observed under field conditions.

For this reason, retrospective data about *APP* isolated from diseased swine in a swine-dense region in the last 14 years is evaluated in this study with respect to antibiotic resistance and coinfections. The most emphasis for prevention of disease with or without vaccination must be the identification and removal of trigger factors including coinfecting agents of risk. The latter are difficult to define, because experimental and field studies with respect to coinfections are rare and in some cases difficult to interpret. Recently, a meta-analysis of porcine respiratory tract coinfections was published, indicating that secondary infection with porcine circovirus 2, and swine influenza virus is aggravating *APP*-related disease, while secondary infection with *APP* can aggravate Swine influenza and *Mycoplasma-hyopneumoniae*-related disease (4). *In vitro* study results suggest that pre-infection with *APP* was able to block the replication of porcine reproductive and respiratory syndrome virus (PRRSV). This antiviral activity was found to be due to production of interferon γ. PRRSV and *APP* coinfection resulted in a stronger cytotoxic effect *in vitro* compared to monoinfection (5). In *in vivo* studies, different outcomes were observed in double infection with PRRSV and *APP*, indicating that PRRSV infection is not necessarily a trigger factor for *APP*-related diseases (6). This implies that further factors can be decisive during the course of coinfections. Specific combinations with coinfecting agents are highly variable in their pathomechanisms. PCV2 was found to promote the adhesion, invasion, and survival of *APP* in porcine alveolar macrophages by decreasing proinflammatory and antiviral cytokines (7). *APP* and influenza coinfections resulted in a clear potentiation of lung lesion severity and more severe clinical symptoms (8). Recently, the effects of *S. suis* nuclease degrading porcine neutrophilic extracellular traps and promoting the growth of *APP* have been shown (9).

Coinfection models are restricted to the specific time course and order of infection as well as the use of a specific *APP* strain which is mostly not reflecting the field situation. Up to now, 19 *APP* serotypes have been described which differ in their pathogenicity and might behave differently in a situation of coinfection with other agents (10–12). Serotypes can be differentiated by their capsule synthesis genes (*cps*) (13). Typing is important to choose the most promising vaccine candidate and to stay informed about the occurrence of emerging serotypes in a region. The serovar-dependent Apx toxin pattern is contributing to varying virulence of strains. Apx toxins are cytolytic and hemolytic and cause characteristic fibrinous and necrotic lung alterations within hours (14). Serotypes producing both ApxI and ApxII (1, 5, 9/11, 16) are considered more virulent than those producing ApxII and ApxIII (2, 3, 4, 6, 8, 15), or either ApxI (10, 14) or ApxII (7, 12, 13, 17, 18, 19) alone (14). Next to these specific Apx I–III patterns, all *APP* are characterized by production of ApxIV (15). In most European countries, *APP* ST2 is the dominant strain causing most disease outbreaks (16–18). This was confirmed recently in a German study based on more than 200 *APP* isolates originating from the same geographical area from the years 2010–2019, where 64% were found to belong to ST2 as the predominant serotype (19). Only 6% of these ST2 isolates showed an atypical *apx* toxin gene pattern in that study. Due to the fact that the method of molecular serotyping based on *cps* genes has been developed in the recent years, most isolates from past years have been typed by older methods as agglutination or PCR based on toxin gene patterns. For the most common ST2, the combination of toxin gene typing and agglutination was confirmed to be reliable by later validation using the *cps* gene PCR.

In Germany, antimicrobial usage (AMU) and antimicrobial resistance (AMR) data derive from passive surveillance reportings in the years 2008, 2010, 2012, and 2015. Obligatory monitoring of AMU was enforced in 2014 in the 16th Amendment of the German Pharmaceuticals Act. Since 2011, the AMU in animals in Germany was more than halved and treatment frequency was reduced in swine farms (20–22). In weaners, respiratory diseases were most often treated with antimicrobials, primarily using amoxicillins and tetracyclines (23). Whether the reduction of AMU in swine will also lead to a reduction of bacterial isolates resistant to antimicrobials is expected but can only be hypothesized. Antimicrobial treatment is the last consequence dealing with recurrent respiratory disease caused by *APP*. For this reason, data on susceptibility of *APP* strains originating from 2006 until 2020 against most important antimicrobial substances used in swine were evaluated retrospectively in this study. A risk of treatment failures in the future should be assessed. In addition, the high impact of coinfecting agents as triggers of disease is known, so that the frequency of coinfections was also evaluated in this study.

## Materials and Methods

### Sample Collection, Isolation of Bacterial Strains, and Susceptibility Testing

Dead or alive pigs were sent for routine diagnostics to the Field Station for Epidemiology of the University of Veterinary Medicine Hannover, Germany, when they suffer from clinical signs of disease or originate from a farm with recurrent disease.

Diagnostic data from pigs with lung diseases sent for necropsy in the time period from January 1, 2006, to December 31, 2020, were screened for successful isolation of *APP* from the respiratory tract. In total, a dataset of 1,680 animals positive for *APP* was generated. In some animals, in addition to the respiratory tract other organ alterations were found and sampled. During the procedure of routine diagnostics, further diagnostic steps were prioritized according to the anamnestic report and the macroscopic findings. Therefore, not all pigs included in the evaluation had been examined for the same panel of pathogens. The study protocol was reviewed by the Animal Welfare Officer and corresponding legal entities before the start of the study (TVO-2019-V-29). Thereby, it was determined that the study did not require permission under the German legislation on animal testing. The study was based on retrospective evaluation of data originating from animals sent for routine diagnostics.

Overall, *APP* isolates originated from 1,243 farms in a swine-dense region in North Western Germany. Most farms were located in neighboring zones around the diagnostic lab in the northwestern part of Lower Saxony. Information about the age group of the pigs was available only retrospectively, but most samples originate from nursery and fattening pigs due to the fact that these age groups are most often affected by porcine pleuropneumonia.

During necropsy, one sample from a lung main bronchus and one sample from affected lung tissue were harvested routinely for bacteriological examination. In case that lung lesions were suspicious for *APP* (necrotic or fibrinous pneumonia), samples were taken from these parts. In addition, a piece of lung tissue was either immediately examined by PCR for respiratory tract pathogens or stored at −20°C for further diagnostics by PCR. Other organ sites were sampled in case of pathological organ alterations.

### Bacterial Cultivation and Isolation of *A. pleuropneumoniae*

Routine cultural diagnostics of bacterial pathogens from lung tissue and other organs followed the standard operating procedures for clinical veterinary microbiology in our accredited laboratory (24, 25). In total, 1,680 isolates were included in the evaluation. Lung tissue and bronchial and pleural swabs were plated on four culture plates, namely, chocolate blood agar containing nicotinamide adenine dinucleotide (NAD, Blood Agar No. 2, Becton, Dickinson and Company, Sparks, NV, USA) for culture of *Pasteurellaceae* and *Alcaligenaceae* (e.g., *Bordetella* spp.), Columbia agar with 5% sheep blood (Becton, Dickinson and Company, Sparks, NV, USA), Gassner agar (Oxoid, Hampshire, UK), and CNA blood agar (Becton, Dickinson and Company, Sparks, NV, USA) containing polymyxin E and nalidixic acid for selective culture of *Staphylococcus* spp. and *Streptococcus* spp. (25–27). Inoculated plates were incubated for 48 h at 37°C under standard atmospheric conditions, while chocolate blood agar was incubated in an 8% CO_2_ atmosphere. Plates were inspected after 24 and 48 h (24). For further typing by their cultural and biochemical properties, single bacterial colonies were subcultivated.

Colonies resembling *APP* were subcultivated on chocolate blood agar and tested biochemically for urease, catalase, and the CAMP phenomena following routine diagnostic protocols (24, 25). Pure cultures of *APP* were stored at −80°C in Cryobank (Cryobank, Mast Group Ltd., Bootle, UK). In most cases, *APP* was confirmed by PCR (see Detection of pathogens by PCR). In the time period 2006–2016, colony material was mixed with specific antisera for serotyping by slide agglutination (Porcs Reactif coagglutine, BioVac, Beaucouzé Cedex, France) (28). Since 2016, *APP* was in addition toxin-typed by PCR diagnostics in accordance with the method of Rayamajhi et al. (29).

### Detection of Pathogens by PCR

For direct detection of pathogens in lung tissue, 2 × 25-mg tissues were extracted using either a commercially available DNA (DNeasy Blood & Tissue Kit, Qiagen GmbH, Hilden, Germany) or an RNA extraction kit (RNeasy Blood & Tissue Kit, Qiagen GmbH, Hilden, Germany). After extraction, 2.5 μl template DNA was added to the PCR master mix composed of 1.5 μl water, 5 μl DNA or RNA Reaction Mix, and the respective assay components following the manufacturer's instructions. Amplification was performed in a real-time cycler with pathogen-specific amplification protocols (Applied Biosystems 7500 Real-Time PCR System, Thermo Fisher Scientific, Waltham, MA, USA). For detection of specific genome fragments of *M. hyopneumoniae*, a multiplex real-time PCR was used (30). DNA fragments of *M. hyorhinis* were detected by a commercially available real-time PCR (BactoReal® Kit *Mycoplasma hyorhinis*, Ingenetix GmbH, Vienna, Austria) according to the manufacturer's instructions as described elsewhere (31). PCV2-specific genome fragments were examined by a TaqMan-based real-time PCR as published elsewhere (32). PRRSV 1 and 2 as well as influenza A were detected by commercially available PCR diagnostic kits following the manufacturer's instructions. The PRRSV RT-PCR allowed the differentiation of PRRSV-1 and−2 (EZ-PRRSV™ MPX 4.0 assay, Tetracore®, Rockville, MD, USA) and is routinely used in diagnostic samples (33). The influenza A RT-PCR (EZ-Universal Flu A 2.0 RT-PCR, Tetracore®, Rockville, MD, USA) was designed by the manufacturer to detect all known subtypes in swine (34).

### Antimicrobial Susceptibility Resting

An important diagnostic step is the determination of the minimal inhibitory concentration (MIC) of different antimicrobial substances to support the veterinarian in decision making for the most effective antimicrobial substance to treat porcine pleuropneumonia on the respective farm. Antimicrobial susceptibility testing of *APP* for thirteen antimicrobial agents of different concentrations followed the recent Clinical Laboratory Standards Institute (CLSI) manuals in routine diagnostic methods (35–40). The method was described in a recent study (25). Resistance testing and interpretation of MICs followed the different guidelines of the CLSI (40–42). Briefly, the optical density of a suspension of colony material in 5 ml 154 mM NaCl was adjusted to McFarland 0.5 in a densitometer (bioMérieux Marcy l'Etoile, Marcy l'Etoile, France), which corresponds to 10^6^-10^8^ CFU/ml (43). Broth microdilution testing of APP is described in the respective CLSI guideline (36). Ten ml of sterile Haemophilus Test Medium bouillon (HTM Broth Thermo Scientific Sensitive™, Thermo Fisher Scientific, WA, USA) was mixed with 50 μl of the suspension. Subsequently, 50 μl of the suspension was pipetted to each well of a commercially available microtiter plate (Sensititre® NLV 39, TREK Diagnostic Systems Ltd., Cleveland, OH, USA) followed by incubation at 35+2°C, 5% CO_2_, for 20–24 h. Wells of the microtiter plate are coated with antimicrobial substances in a two-fold dilution series as provided by the manufacturer and suggested by the German Veterinary Society working group on AMR (39): ampicillin (0.12–32 mg/l), ceftiofur (0.12–8 mg/l), cephalothin (1–16 mg/l), enrofloxacin (0.03–2 mg/l), florfenicol (1–8 mg/l), gentamicin (0.25–16 mg/l), penicillin G (0.06–16 mg/l), spectinomycin (4–64 mg/l), tetracycline (0.12–16 mg/l), tiamulin (8–32 mg/l), tilmicosin (1–32 mg/l), and tulathromycin (2–64 mg/l). The MIC of the different substances was determined for each *APP* isolate. Interpretation of growth inhibition followed the clinical breakpoints approved by the CLSI (35, 37, 38). If no approved clinical breakpoints are available, other published clinical breakpoints used in established routine evaluation procedures were used for assessment. For cephalothin, the clinical breakpoint for streptococci in dogs (8 mg/l) was used (40). For penicillin G, the clinical breakpoint for *Pasteurella multocida* in swine (0.25 mg/ml) and for spectinomycin for *Pasteurella multocida* in cattle (32 mg/ml) was used (40). *APP* isolates were determined to be “resistant” (r), “intermediate” (i), or “susceptible” (s) according to their clinical breakpoints (25, 39).

### Data Management and Statistical Evaluation

All laboratory data were registered by means of the laboratory information system Lab Control© 2002, Ticono-Software, Hannover, immediately after receipt of test results. Prior to statistical evaluation, all data were exported to Excel, Version 2010 (Microsoft Corporation, Albuquerque, NM, USA), for data management. Statistical analyses were performed *via* SAS, version 9.4 (SAS Institute, Cary, NC, USA).

Only the first *APP* isolate from a respective farm within 1 year was included in the final data set to guarantee independent samples for the statistical evaluation. Finally, the data set was based on 1,680 App isolates originating from 1,243 farms. Different *APP i*solates from one farm were considered as independent, because at least a 1-year timely distance was realized between two recordings. Coinfections were categorized according to the number of detected coinfecting agents and the distribution of coinfection patterns.

Data were described following usual calculations with proportions, means, and graphical presentations. For further evaluation of a time-related trend in the development of antimicrobial resistance, two time periods 2006–2014 and 2015–2020 were compared. The first time period represents the reference period, while the second time period was characterized by stricter legislation with respect to prescription of antimicrobials since 2011 and the implementation of the German national antibiotic database in 2014. As a consequence in this time period, a significant reduction of AMU in swine production in Germany was achieved (21, 44–46). To address the hypothesis of an impact of reduction in AMU on frequencies of resistant *APP* isolates, frequencies were compared by means of univariable and multivariable logistic regression models taking different sampling sites and time periods into account.

The development of the proportion of *APP* serotype 2 over time was evaluated in a linear logistic regression model. The level of significance for all statistical models was set at 0.05 without any adjustment for multiple testing.

## Results

### Microbiological Findings and Meta-Data for *A. pleuropneumoniae*

In the time period 2006–2020 in total, 1,680 samples out of 1,243 farms were found to be positive for *APP* by bacteriological culture. Seventy three percentage of the isolates originated from farms in the neighborhood of the diagnostic lab. In 655 pigs (39%), the age group was recorded resulting in 2.9% of isolates originating from sows, and 1.2% of isolates originating from suckling piglets and 56 and 40% originating from nursery and fattening pigs, respectively. The majority of strains (94%) were isolated from the lower respiratory tract or pleural cavity (4%). In rare cases (2%), *APP* was detected in other less defined organ samples by the same cultural diagnostic procedure as described above, such as in bronchoalveolar lavage fluid (*n* = 4), abscesses (*n* = 6) and swabs from tonsil, nose, and serosa (*n* = 7). Sixteen isolates originated from the abdominal cavity, brain, or joints (0.95%).

**Figure 1:**
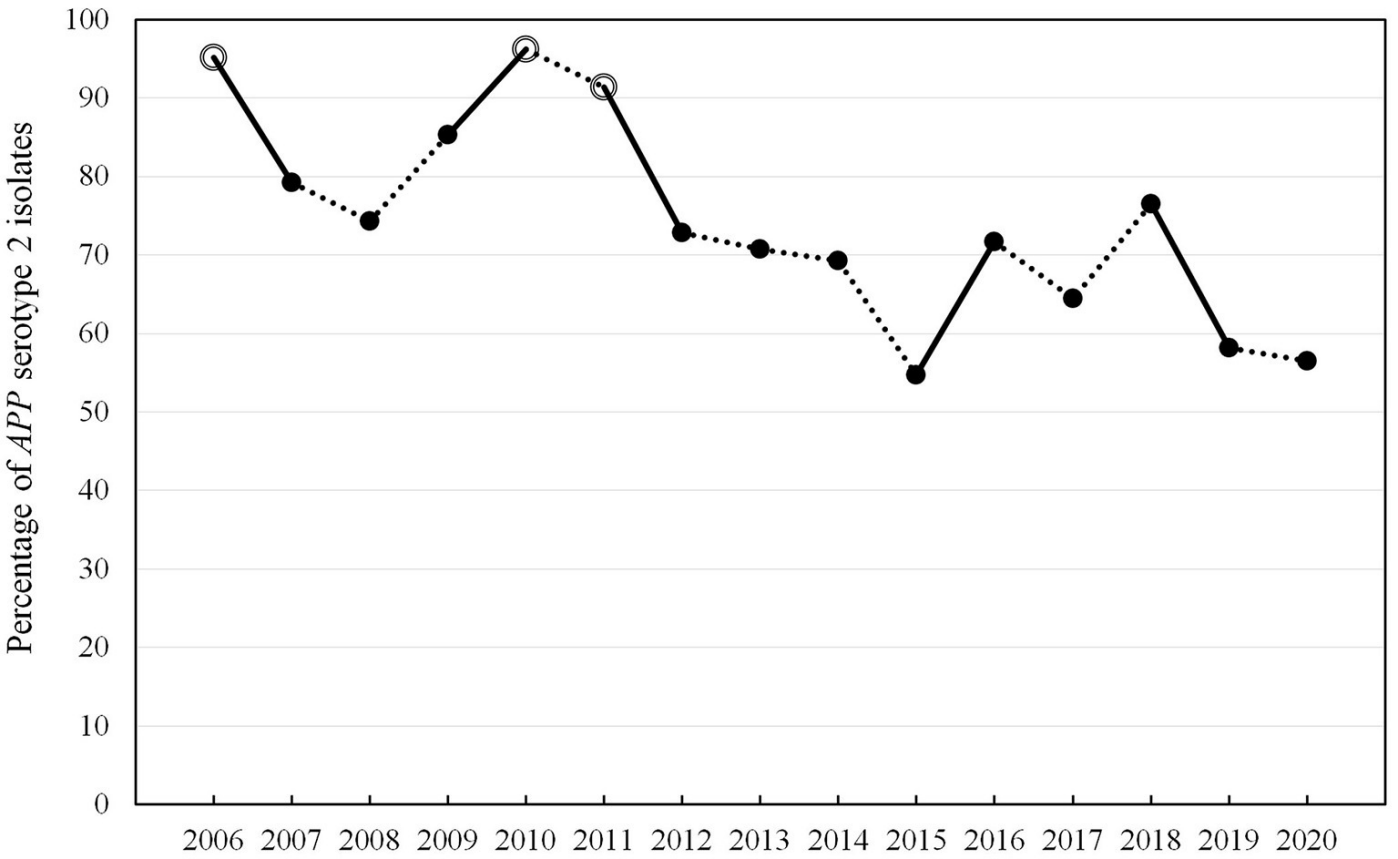
Percentage of *APP* serotype 2 isolates in the years 2006–2020 determined by slide agglutination with specific rabbit antisera (strict lines show statistical significant changes from 1 year to the next, white dots indicate no significant changes to the initial year 2006, while black dots indicate significant changes compared to 2006 with *p* < 0.05 in Wald test *via* linear logistic regression).

In the whole time-period, different typing methods were used to define the respective serotype of the isolates due to the ongoing technological progress in the spectrum of method development over time. In total, 76% (*n* = 1,276) of the isolates were analyzed further for the serotype. Out of the typed isolates, *APP* serotype (ST) 2 was detected most often (*n* = 955, 75%). In the time period prior to 2016, no typing for the toxin gene pattern of *APP* was possible and only slide agglutination with specific antisera was performed, which is of lower specificity than PCR typing. For this reason, evaluation of the percentage of serotypes determined by the toxin gene pattern was only performed in the time period 2016–2020. The percentage of *APP* ST2 was between 56 and 74% in this time period (Table 1). Out of 397 isolates, 46 were not typable by PCR based on the toxin gene pattern (11.6%) (29). Determination of ST2 by slide agglutination with rabbit antisera specific for this serotype was found to be reproducible and specific in comparison to PCR results since 2016. Due to the fact that it has been used in the past since 2006 as a routine method, the whole data set from the years 2006 to 2020 was evaluated with respect to ST2 frequencies detected over time (Table 2, Figure 1) (47–49). Proportions of ST2 isolates fluctuate between subsequent years. In total, a statistically significant decrease in percentage of ST2 was found from 2006 to 2020 (*p* < 0.0001).

**Table 1:** Serotype distribution of 351 typable *A. pleuropneumoniae* isolates defined by toxin gene pattern and slide agglutination in the years 2016–2020.

*APP* serotype^*^	**2016**		**2017**		**2018**		**2019**		**2020**		**Total**	
	***n***	**%**	***n***	**%**	***n***	**%**	***n***	**%**	***n***	**%**	***n***	**%**
1	1	1.7	1	1.5	-	0.0	2	2.3	4	6.5	8	2.3
2	43	74.0	49	74.0	65	82.3	50	58.1	35	56.5	242	68.9
4	-	0.0	-	0.0	-	0.0	1	1.2	-	0.0	1	0.3
5	4	6.9	4	6.0	2	2.5	-	0.0	1	1.6	11	3.1
6	-	0.0	1	1.5	2	2.5	2	2.3	2	3.2	7	2.0
7	2	3.4	1	1.5	-	0.0	2	2.3	3	4.8	8	2.3
8	-	0.0	1	1.5	-	0.0	16	18.6	8	12.9	25	7.1
9	8	13.8	9	13.6	8	10.1	12	14.0	9	14.5	46	13.1
12/13	-	0.0	-	0.0	2	2.5	1	1.2	-	0.0	3	0.9
Total	58	16.5	66	18.8	79	22.5	86	24.5	62	17.7	351	100

* *Only strains with concordant findings in toxin gene PCR and agglutination were recorded in this table*.

**Table 2:** Outcome of linear logistic regression model for comparison of proportion of *APP s*erotype 2 in subsequent years.

**Years compared with respect to proportion of *APP* ST2**	**OR**	**Univariable model 95% CI**		***p***
		**Lower**	**Upper**	
**2007 vs. 2006**	**0.241**	**0.075**	**0.775**	**0.0170**
2008 vs. 2007	0.652	0.290	1.467	0.3013
2009 vs. 2008	2.207	0.975	4.995	0.0576
**2010 vs. 2009**	**3.702**	**1.006**	**13.631**	**0.0490**
2011 vs. 2010	0.850	0.184	3.921	0.8350
**2012 vs. 2011**	**0.138**	**0.047**	**0.408**	**0.0003**
2013 vs. 2012	0.932	0.537	1.618	0.8034
2014 vs. 2013	0.865	0.451	1.662	0.6640
2015 vs. 2014	0.618	0.301	1.270	0.1906
**2016 vs. 2015**	**2.259**	**1.031**	**4.951**	**0.0418**
2017 vs. 2016	0.644	0.291	1.425	0.2775
2018 vs. 2017	1.695	0.825	3.481	0.1507
**2019 vs. 2018**	**0.385**	**0.196**	**0.756**	**0.0056**
2020 vs. 2019	0.969	0.499	1.883	0.9265

*One-factorial logistic regression model with fixed effect time with respect to percentage of APP serotype 2 isolates. OR: point estimate/odds ratio, p: p-value of the Wald test, 95% CI: upper and lower confidence limits of the 95% confidence interval of the Wald test. Significant changes are indicated in bold*.

### Distribution of Antimicrobial Resistance

The lowest frequencies of resistant isolates were found for cephalothin, ceftiofur (0.18%), florfenicol (0.24%), tilmicosin (2.4%), tiamulin (2.3%), enrofloxacin (2.7%), and spectinomycin (3.5%). About 6% of all isolates were resistant to amoxicillin. The highest frequencies of resistant isolates were found for gentamicin (31.1%), penicillin (51.8%), and tetracycline (78.3%). The distribution of MICs of respective *APP* isolates is depicted in Table 3.

**Table 3:** MIC distribution of *APP* isolates originating from the respiratory tract and the pleural cavity tested within the time period 2006–2020.

**Substance [clinical breakpoint (mg/L)]**		**Number of isolates with MIC-values (mg/L) of…**												**MIC_**50**_**	**MIC_**90**_**	**s**	**i**	**r**
	**n**	**≤0.03**	**0.06**	**0.12**	**0.25**	**0.5**	**1**	**2**	**4**	**8**	**16**	**32**	**≥64**	**(mg/L)**		**%**	**%**	**%**
Ampicillin (≤0.5)	1647			1267	273	12	2		2	7	15	28	41	≤0.12	0.25	94.23	0.12	5.65
Ceftiofur (≤2)	1647			312			1326	6	1		2			1.00	1.00	99.82	0.06	0.12
Cephalothin^a^ (≤8)	1647						299	9	1327	9	3			4.00	4.00	99.82	0.18	0
Enrofloxacin (≤0.25)	1647	260	1249	52	41	32	12	1						0.06	0.06	97.27	1.94	0.79
Florfenicol (≤2)	1647						1600	43	2	2				≤1.00	≤1.00	99.76	0.12	0.12
Gentamicin (≤2)	1647				7	8	36	1084	470	39		3		2.00	4.00	68.91	28.54	2.55
Penicillin G^b^ (≤0.25)	1646		283	72	438	705	49	5	2		79	13		0.50	0.50	48.18	42.83	8.99
Spectinomycin^c^(≤32)	1647								4	74	790	722	57	16.00	32.00	96.54	2.25	1.12
Tiamulin (≤16)	1645								276	813	518	18	20	≤8.00	16.00	97.69	0.00	2.31
Tilmicosin (≤16)	1647						2	7	445	1030	124	10	29	8.00	8.00	97.63	0.00	2.37
Tetracycline (≤0.5)	1647			4	24	329	1047	34	14	80	49	66		1.00	8.00	21.68	63.57	14.75
Tulathromycin^d^ (≤64)	312							8	42	172	82	8		8.00	16.00	100	0.00	0.00

*Susceptibility and resistance of A. pleuropneumoniae isolates were assessed with respect to clinical breakpoints of the CLSI standards for A. pleuropneumoniae in swine. Bacterial isolates were categorized as “susceptible” (s), “intermediate” (i), or “resistant” (r). If no CLSI-approved clinical breakpoints for APP in swine were available, other clinical breakpoints published in CLSI VET01S ED5:2020 were used for assessment:*

a *streptococci in dogs*,

b *Pasteurella multocida in swine, and*

c *Pasteurella multocida in cattle*.

d *Tulathromycin was tested since 2017. The white area contains the dilution ranges tested. When isolates grew in the highest concentration of an antimicrobial agent, the corresponding MICs are considered to be equal to or higher than the next (not tested) concentration*.

The sixteen isolates originating from sites outside the respiratory tract were fully susceptible for ceftiofur, cephalothin, enrofloxacin, florfenicol, spectinomycin, tiamulin, and tulathromycin. Resistances were found in 25% (4/16) isolates for ampicillin and gentamicin, 37.5% (6/16) for penicillin, 6.25% (1/16) for tilmicosin, and 31.25% (5/16) for tetracycline. Interquartile ranges of MICs of the tested antimicrobials were narrow due to the mainly high susceptibility rate. One dilution step was the highest interquartile range in MICs found for gentamicin (2–4 mg/l), spectinomycin (16–32 mg/l), tiamulin (8–16 mg/l), and tulathromycin (8–16 mg/l).

### Temporal Trends in AMR

**Figure 2:**
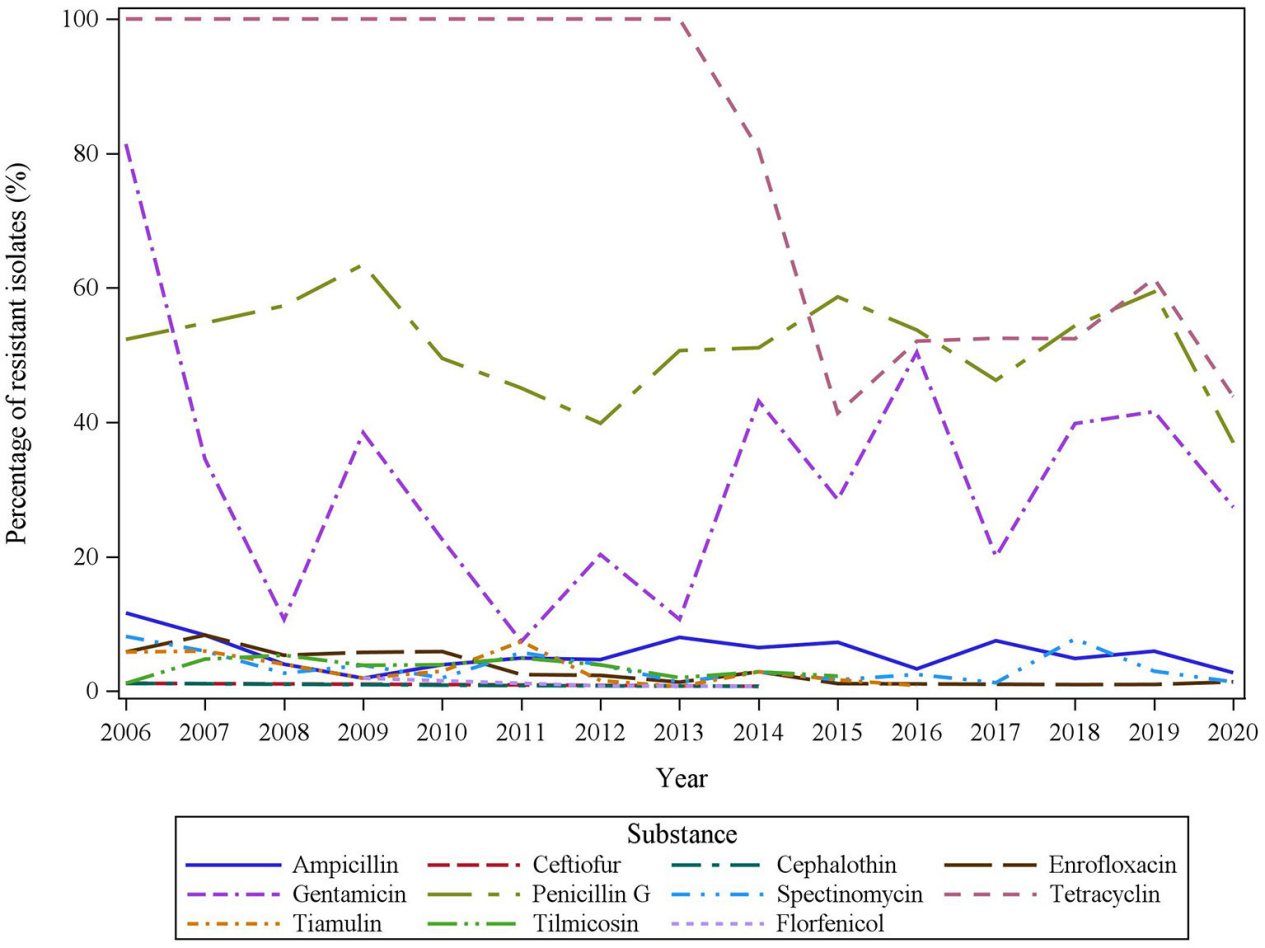
Time dependent development of resistant isolates in the years from 2006 to 2020.

The frequencies of *APP* isolates resistant to different substances were compared between the different years in the time period 2006 to 2020 for 1,647 isolates. Isolates originating from organ sites outside the respiratory tract (*n* = 33) were excluded from this evaluation. The development over time is shown in Figure 2. For statistical comparison, the group of isolates being intermediate or resistant to a respective substance according to the clinical cutoff as shown in Table 3 was combined as the group of resistant isolates. No differences in frequencies of resistant isolates were found between different age groups in the set of data with known age group (data not shown).

The relative frequency of resistant *APP* isolates in the time period 2006–2020 is shown in Figure 2, showing inconsistent findings with respect to AMR within different years.

We addressed the hypothesis that a consequent reduction in antibiotic usage in swine production since 2014 had an influence on frequencies of bacterial resistance; the two time periods from 2006–2014 to 2015–2020 were compared in uni- and multivariable logistic regression models. Logistic regression model outcomes for different substances are shown in Supplementary Table 1. For cephalothin, ceftiofur, and florfenicol, the models were not valid due to the low number of resistant isolates. No differences were found between the respiratory tract and the pleural cavity with respect to frequency of resistant isolates in the different time periods. For enrofloxacin, tiamulin, and tilmicosin, significantly lower frequencies of resistant isolates were found in the last 6 years (enrofloxacin: 4.04% 2006–2014 vs. 0.8% in 2015–2020, *p* = 0.0004, tiamulin: 3.4% in 2006–2014 vs. 0.6% in 2015–2020, *p* = 0.0009, tilmicosin: 3.5% 2006–2014 vs. 0.6% in 2015–2020, *p* = 0.0007). A more pronounced difference between the two time periods was found for tetracycline (2006–2014: 97.3% vs. 2015–2020: 49.8%, *p* < 0.0001). In contrast to that, an increase in gentamicin-resistant isolates was found in the last 6 years compared to 2006–2014 (28.4 vs. 35.2% in 2015–2020, *p* = 0.0037).

### Coinfecting Agent

All animals positive for *APP* were tested for other coinfecting agents in the respiratory tract depending on either anamnestic clinical or postmortal pathomorphological findings during the routine diagnostic procedures. The frequencies of coinfecting pathogens are shown in Table 4.

**Table 4:** Coinfecting pathogens in the respiratory tract of pigs positive for *APP*.

**Coinfecting pathogen**	**Number of samples tested**	**Negative (%)**	**Positive (%)**
Influenza virus A^a^	423	90.1	9.9
PRRSV EU^a^	489	76.7	23.3
PRRSV US^a^	486	90.5	9.5
Porcine Circovirus 2^a^	109	44.0	56.0
M. hyopneumoniae^a^	420	80.5	19.5
M. hyorhinis^a^	263	87.5	12.5
Bordetella bronchiseptica^*^	1680	97.0	3.0
Glaesserella parasuis^*^	1678	96.5	3.5
Pasteurella multocia^*^	1680	85.5	14.5
β-Hemolytic streptococci^*^	1680	85.5	14.5
Staphylococcus aureus^*^	1680	98.3	1.7
Streptococcus suis^*^	1680	75.2	24.8

a *Detection by PCR*,

* *detection by cultural microbiological methods*.

A large proportion of samples were only positive for *APP* as the only pathogen, although samples had been tested for other pathogens (50.1%). In total, 132 combinations of various pathogens were found, which were dominated by combinations with *S. suis* alone (8.5%) or with additional other bacterial or viral pathogens (16.3%). Combinations with PCV2, PRRSV 1, influenza A virus, and in addition any other viral or bacterial pathogen in the tested samples were 4.6, 15, and 8.0%, respectively. A maximal combination of six different pathogens including *APP* was found in one sample.

## Discussion

In this study, routine diagnostic data from swine with respiratory diseases in a swine-dense region in North Western Germany were evaluated with respect to the primary respiratory tract pathogen *APP*. All isolates were harvested between 2006 and 2020, and the data set was restricted to one isolate from one farm per year. Approximately 1% of the isolates originated from body sites outside the respiratory tract, as brain and joints. Spreading of *APP via* lymphatic vessels or the blood stream during a period of septicaemia has recently been examined under experimental conditions (50). In weaners infected by aerosol with *APP* serotype 7, the pathogen was isolated frequently in inner organs within the first week after infection. Approximately 15% of the animals were positive for the pathogen in the joints and about 28% in the central nervous system. Isolation of *APP* from peripheral tissue was correlated with spleen colonization and might be the consequence of spreading *via* the lymph system. It can be expected that septicaemia occurs in a high proportion of animals in the acute stage of infection, which will not be detected at a later point of time, when diseased or died pigs were sent for necropsy. In a field study, fibrinopurulent arthritis and necrotizing osteomyelitis were found to be caused by an *APP* serotype 2 isolate in 8–12-week-old pigs. The pathogen inside the pathological organ alterations was successfully verified by fluorescence *in-situ* hybridization in that case report (51). In addition, the examined pigs suffered from a chronic fibrinonecrotizing pleuropneumoniae and adhesive pleuritis. It can be assumed that inner organ spreading occurs as sequelae to pleuropneumoniae in some cases. In our data set, the serotype of eight *APP* isolates originating from joints or the central nervous system could be determined as serotype 2 (*n* = 5), serotype 9 (*n* = 2), or serotype 7 (*n* = 1).

As expected, *APP* serotype 2 was most prevalent within the data set with 76%. In the time period 2016–2020, the proportion of serotype 2 was 61%, which is in accordance with the findings of Schuwerk et al. (19) who serotyped 213 *APP* from the same geographic region in the years 2010–2019 and found 64% serotype 2 (19). Surprisingly, they described an increase in this serotype since 2010, but data were not statistically evaluated. Also, the other serotypes were found in similar proportions with 15% serotype 9/11 but also 4% of non-typable isolates. In more than 93% of the isolates, the toxin profile was found to be typical for the respective serotype, which was confirmed by the gold standard for *APP* serotyping, the comprehensive typing of the capsular polysaccharide loci by a multiplex PCR (13). By several authors, the occurrence of *APP* with atypical *apx* toxin gene profiles was reported, so that capsular typing is considered as the reference method. The validity of the serovar-specific association of the toxin genes was checked recently, resulting in approximately 7% of isolates with an atypical toxin gene pattern. Six percentage of the serotype 2 isolates and several non-typable and serotype 18 isolates were found with toxin profiles deviating from the general pattern (19). Therefore, there might be a bias in our study in case that atypical serotype 2 isolates were missed due to methodic reasons. In case that 6% atypical serotype 2 isolates were missed, the proportion of serotype 2 isolates in 2016–2020 would increase to 67%. No differences between the serotypes could be found with respect to antibacterial resistance (data not shown).

In general, secondary data from routine diagnostics as used in the present analysis are prone to bias mainly due to the lack of metadata and the non-standardized sampling of diseased animals. Although approximately half of the German swine population is located in this region, samples cannot be considered representative of the whole of Germany. A drawback of this evaluation is the lack of meta-data, so that potential confounding effects on antimicrobial resistance might be masked with AMU on respective farms being the most important. Nevertheless, the significant trends observed in resistance development from a routine data pool even under these conditions might indicate a relevant phenomenon of practical impact.

In *Pasteurellaceae*, resistance genes can be distributed by conjugative and integrative elements, plasmids, and transposons, so that monitoring of antimicrobial resistance in clinical isolates is recommended to detect any negative developments as soon as possible (52).

Established clinical breakpoints based on achievable tissue concentrations after treatment with the respective substance were used to assess a temporal development of antimicrobial resistance. The underlying hypothesis was that a reduction in antibiotic usage in this region is accompanied by a reduction in frequencies of resistant isolates over time. The significant changes in frequencies of resistant isolates can be assigned to the time period, because evaluation with respect to serotype, sampling site, or age group in a subset of data did not reveal any significant differences (data not shown). The obtained MIC_50_ and MIC_90_ values in this data set were comparable to those from 2009 to 2012 published within the European VetPath program in 2016 (53). By contrast, MICs for ceftiofur were slightly above those published, while MICs for macrolides were lower in this study. The high effectivity of one single injection of a macrolide (gamithromycin) and also two administrations of florfenicol has been shown under field conditions (54). The high efficacy of fluoroquinolones was recently shown in defined experimental *APP* ST2 challenge experiments (55). A single injection of marbofloxacin or three consecutive injections of enrofloxacin were found to be successful for treatment of an acute pleuropneumonia. Quinolones were assessed to be effective also in other studies monitoring clinical *APP* isolates for antimicrobial resistance (56, 57). Nevertheless, *APP* strains resistant to quinolones sporadically occur and several mechanisms such as a decrease in drug influx mediated by downregulation of specific outer membrane proteins, overexpression of an efflux pump, and a less effective drug binding have been described recently (58). Exposure of *APP* to subinhibitory concentrations of ciprofloxacin was found to increase the MIC up to 2 μg/ml in a lab experiment. It was shown that a cross-resistance to ampicillin can develop in parallel to a ciprofloxacin resistance. Ciprofloxacin-resistant mutant strains differed in several phenotypic characteristics from wild-type strains, also negatively affecting their bacterial fitness (59). This might be the reason for the so far low prevalence of quinolone-resistant strains observed in the field. In contrast to that, the proportion of tetracycline-resistant isolates was high, but even higher levels of resistance to tetracyclines have been reported for Canadian isolates in 2012 (57). Except for macrolides, antimicrobial resistance genes of *APP* were found to be correlated with phenotypic AMR profiles (40). In that study, the proportions of resistant isolates from UK were lower for tetracycline (57%), but higher for enrofloxacin (6%) and amoxicillin (20%) compared to the findings in our study.

Although no parallel data evaluation on AMU and antimicrobial resistance on farms was performed, the effects of reduction of antibiotic usage in this swine-dense region might be reflected. The reported time-dependent analysis of antimicrobial resistances in *APP* resulted in a significant decrease in the frequency of isolates resistant to enrofloxacin, tiamulin, tilmicosin, and tetracycline compared to the time period 2006–2014. These decreases are in parallel to a reduction in antibiotic usage in livestock in Germany since 2011 reflected by a reduction of the administered daily doses (20, 21). A significant reduction in treatment frequencies were already recorded within the German VetCAb in 2014 in piglet fattening (21, 23, 60). The German Pharmaceutical Act was strengthened in 2014, and treatment frequencies were reported continuously in the German Quality assurance database. Although aminoglycoside antibiotics were also reduced by more than 36% in 2011–2018 (22), antimicrobial resistance in *APP* to gentamicin increased over time in our data set. It has to be taken into account that additional factors have a significant influence on antimicrobial resistance on herd level, time span between sampling and treatment, distance to other farms, space allowance, and cleanliness (61).

Important preventive measures against outbreaks due to *APP* and the subsequent treatment with antimicrobials include vaccination, avoidance of mixing of pigs, and prevention of any trigger factors as specific coinfections. Although three commercial vaccines are available in Germany and autologous bacterin vaccines are also widely used, vaccination is often not successful. One commercial subunit vaccine based on Apx toxins confers cross-protection against all serotypes, while the other two bacterin vaccines contain either serotypes 1 and 2 or serotypes 2 and 9 providing protection against the most prevalent serotype 2 and other serotypes. Prevention of the disease by vaccination is mainly hampered by a lack of an adequate mucosal and cellular immunity, while innate immunity is most decisive for the course of the disease (62). Any factor weakening the mucosal immune system can initiate a disease outbreak (63). Disease outbreaks are mainly triggered by external factors in already endemically infected herds and less by introduction of new *APP* strains (64). The lack of knowledge about the impact and interaction of various trigger factors for a disease outbreak on farms is considered as one major gap in *APP* research so far (65). Isolates from the diseased pigs in this study belonging to serotypes 2, 4, 6, and 8 might be primarily colonizers of carrier pigs more than producing acute and sudden outbreaks, because they were assigned to be moderate pathogenic due to a lack of the Apx I. Of high importance for disease outbreaks caused by the highly prevalent serotype 2 are therefore other trigger factors. In general, a higher microbial species diversity and richness were found to be beneficial for the respiratory health status of pigs (66). The well-adapted upper respiratory tract microbial communities are considered to limit colonization of less-adapted, more pathogenic microorganisms by competitive exclusion (67, 68). Less-adapted, pathogenic microorganisms can be a source of infection after translocation to the lungs. So far, bacteria in the lungs of healthy individuals are considered not to proliferate after microaspiration (neutral dispersal model), because they are cleared before becoming a resident flora (68–70). It can be assumed that in asymptomatic individuals facing a constant bacterial challenge from the upper respiratory tract, subclinical inflammatory and immune regulatory processes in the lung are necessary to maintaining the balance between entry and removal of microorganisms. Any shifts in the microbiome might disrupt this balance in airway clearance (68). Alterations of the delicate balance are categorized to be either the consequence of pulse (e.g., acute infection with new microorganism) or press (long-standing, chronic disturbance, e.g., by immune dysfunction) disturbance. This concept of balance in the respiratory tract should be addressed also in swine medicine to prevent outbreaks of respiratory disease. Coinfecting agents can disturb this balance, which can be hypothesized at least for PCV2, PRRSV, influenza virus, *Pasteurella multocida*, and *Mycoplasma hyopneumoniae* (4, 7, 8, 71).

Also, *S. suis* is often detected in parallel with *APP*. Both organisms are colonizers of the tonsils and were found to interact *in vitro*. *S. suis* DNase has a beneficial effect for *APP* in providing NAD by destruction of extracellular NETs released by neutrophilic granulocytes (9). *S. suis* is a pathobiont in the porcine respiratory tract with high invasive potential (72). Virulent strains can be components of the respiratory microbiome, which become invasive after disruption of the microbiota due to keystone pathogens. Next to *APP* as the target organism causing disease, also coinfecting agents and keystone pathogens must be included in preventive measures. The analysis of the herd-specific host–pathogen–environmental constellation in endemically infected herds has therefore a higher priority than the implementation of a vaccine. Trigger factors found under field conditions must be identified systematically to address the impact of environment next to coinfections and the host's immunity onto the pathogenesis of pleuropneumonia.

## Data Availability Statement

The raw data supporting the conclusions of this article will be made available by the authors, without undue reservation.

## Author Contributions

IH-P designed the study, collected the data, and wrote the paper. JM exported, structured, and checked the data from the laboratory information system. MH and LK analyzed and interpreted the data and reviewed the manuscript. All authors read and approved the final manuscript.

## Conflict of Interest

The authors declare that the research was conducted in the absence of any commercial or financial relationships that could be construed as a potential conflict of interest.

## Publisher's Note

All claims expressed in this article are solely those of the authors and do not necessarily represent those of their affiliated organizations, or those of the publisher, the editors and the reviewers. Any product that may be evaluated in this article, or claim that may be made by its manufacturer, is not guaranteed or endorsed by the publisher.
