# Supplementary material for: Coinfections and Phenotypic Antimicrobial Resistance in Actinobacillus pleuropneumoniae Strains Isolated From Diseased Swine in North Western Germany—Temporal Patterns in Samples From Routine Laboratory Practice From 2006 to 2020
Source: Front Vet Sci. 2022 Jan 28;8:802570. doi: 10.3389/fvets.2021.802570 (PMC8831912; doi:10.3389/fvets.2021.802570)
Supplement: Supplementary file 1 [file Table_1.DOCX]

**Table 1.** Comparison of proportion of resistant *APP* isolates with respect to time period and sampling site.

| Risk categories | resistant | | susceptible | | univariable model | | | | multivariable model | | | |
| --- | --- | --- | --- | --- | --- | --- | --- | --- | --- | --- | --- | --- |
|  | n | % | n | % | OR | 95%-CI | | p | OR | 95%-CI | | p |
|  |  |  |  |  |  | lower | upper |  |  | lower | upper |  |
| Ampicillin | | | | | | | | | | | | |
| 2006-2014 (ref) | 59 | 5.96 | 931 | 94.04 | 1 | x | x | x | 1 | x | x | x |
| 2014-20201 | 36 | 5.48 | 621 | 94.52 | 0.915 | 0.597 | 1.402 | 0.6824 | 0.934 | 0.608 | 1.434 | 0.7541 |
|  |  |  |  |  |  |  |  |  |  |  |  |  |
| Respiratory tract (ref.) | 89 | 5.63 | 1491 | 94.37 | 1 | x | x | x | 1 | x | x | x |
| Pleural cavity | 6 | 8.96 | 61 | 91.04 | 1.648 | 0.694 | 3.915 | 0.2580 | 1.624 | 0.680 | 3.877 | 0.2746 |
| Enrofloxacin | | | | | | | | | | | | |
|  |  |  |  |  |  |  |  |  |  |  |  |  |
| 2006-2014 (ref) | 40 | 4.04 | 950 | 95.96 | 1 | x | x | x | 1 | x | x | x |
| 2014-20201 | 5 | 0.76 | 652 | 99.24 | 0.182 | 0.072 | 0.464 | **0.0004** | 0.184 | 0.072 | 0.470 | **0.0004** |
|  |  |  |  |  |  |  |  |  |  |  |  |  |
| Respiratory tract (ref.) | 42 | 2.66 | 1538 | 97.34 | 1 | x | x | x | 1 | x | x | x |
| Pleural cavity | 3 | 4.48 | 64 | 95.52 | 1.717 | 0.518 | 5.686 | 0.3766 | 1.357 | 0.407 | 4.523 | 0.6189 |
| Gentamicin | | | | | | | | | | | | |
|  |  |  |  |  |  |  |  |  |  |  |  |  |
| 2006-2014 (ref) | 281 | 28.38 | 709 | 281 | 1 | x | x | x | 1 | x | x | x |
| 2014-20201 | 231 | 35.16 | 426 | 231 | 1.368 | 1.107 | 1.690 | **0.0037** | 1.355 | 1.095 | 1.675 | **0.0051** |
|  |  |  |  |  |  |  |  |  |  |  |  |  |
| Respiratory tract (ref.) | 496 | 31.39 | 1084 | 496 | 1 | x | x | x | 1 | x | x | x |
| Pleural cavity | 16 | 23.88 | 51 | 16 | 0.686 | 0.387 | 1.215 | 0.1958 | 0.732 | 0.412 | 1.300 | 0.2869 |
| Penicillin G | | | | | | | | | | | | |
|  |  |  |  |  |  |  |  |  |  |  |  |  |
| 2006-2014 (ref) | 503 | 50.86 | 486 | 49.14 | 1 | x | x | x | 1 | x | x | x |
| 2014-20201 | 350 | 53.27 | 307 | 46.73 | 1.101 | 0.904 | 1.342 | 0.3376 | 1.101 | 0.903 | 1.342 | 0.3437 |
|  |  |  |  |  |  |  |  |  |  |  |  |  |
| Respiratory tract (ref.) | 819 | 51.87 | 760 | 48.13 | 1 | x | x | x | 1 | x | x | x |
| Pleural cavity | 34 | 50.75 | 33 | 49.25 | 0.956 | 0.586 | 1.559 | 0.8571 | 0.976 | 0.597 | 1.594 | 0.9222 |
| Spectinomycin | | | | | | | | | | | | |
|  |  |  |  |  |  |  |  |  |  |  |  |  |
| 2006-2014 (ref) | 38 | 3.84 | 952 | 96.16 | 1 | x | x | x | 1 | x | x | x |
| 2014-20201 | 19 | 2.89 | 638 | 97.11 | 0.746 | 0.426 | 1.306 | 0.3052 | 0.753 | 0.429 | 1.320 | 0.3215 |
|  |  |  |  |  |  |  |  |  |  |  |  |  |
| Respiratory tract (ref.) | 54 | 3.42 | 1526 | 96.58 | 1 | x | x | x | 1 | x | x | x |
| Pleural cavity | 3 | 4.48 | 64 | 95.52 | 1.325 | 0.404 | 4.352 | 0.6423 | 1.252 | 0.379 | 4.131 | 0.7123 |
| Tetracyclin | | | | | | | | | | | | |
|  |  |  |  |  |  |  |  |  |  |  |  |  |
| 2006-2014 (ref) | 963 | 97.27 | 27 | 2.73 | 1 | x | x | x | 1 | x | x | x |
| 2014-20201 | 327 | 49.77 | 330 | 50.23 | 0.028 | 0.018 | 0.042 | **<.0001** | 0.027 | 0.018 | 0.041 | **<.0001** |
|  |  |  |  |  |  |  |  |  |  |  |  |  |
| Respiratory tract (ref.) | 1233 | 78.04 | 347 | 21.96 | 1 | x | x | x | 1 | x | x | x |
| Pleural cavity | 57 | 85.07 | 10 | 14.93 | 1.604 | 0.811 | 3.174 | 0.1747 | 0.657 | 0.272 | 1.585 | 0.3499 |
| Tiamulin | | | | | | | | | | | | |
|  |  |  |  |  |  |  |  |  |  |  |  |  |
| 2006-2014 (ref) | 34 | 3.44 | 955 | 96.56 | 1 | x | x | x | 1 | x | x | x |
| 2014-20201 | 4 | 0.61 | 652 | 99.39 | 0.172 | 0.061 | 0.488 | **0.0009** | 0.179 | 0.063 | 0.509 | **0.0012** |
|  |  |  |  |  |  |  |  |  |  |  |  |  |
| Respiratory tract (ref.) | 34 | 2.15 | 1545 | 97.85 | 1 | x | x | x | 1 | x | x | x |
| Pleural cavity | 4 | 6.06 | 62 | 93.94 | 2.932 | 1.009 | 8.519 | 0.0481 | 2.332 | 0.797 | 6.827 | 0.1222 |
| Tilmicosin | | | | | | | | | | | | |
|  |  |  |  |  |  |  |  |  |  |  |  |  |
| 2006-2014 (ref) | 35 | 3.54 | 955 | 96.46 | 1 | x | x | x | 1 | x | x | x |
| 2014-20201 | 4 | 0.61 | 653 | 99.39 | 0.167 | 0.059 | 0.473 | **0.0007** | 0.167 | 0.059 | 0.473 | **0.0008** |
|  |  |  |  |  |  |  |  |  |  |  |  |  |
| Respiratory tract (ref.) | 37 | 2.34 | 1543 | 97.66 | 1 | x | x | x | 1 | x | x | x |
| Pleural cavity | 2 | 2.99 | 65 | 97.01 | 1.284 | 0.303 | 5.440 | 0.7347 | 1.006 | 0.236 | 4.289 | 0.9930 |

Logistic regression analysis with fixed effect time period and factor “sampling site” with respect to different antimicrobial substances. Reference categories for the logistic regression method (time period 2006-2014, sampling site respiratory tract) is indicated by “Ref.”. Univariable model: One-factorial logistic regression model, Multivariable model: Multi-factorial logistic regression model, OR: Point estimate /Odds ratio, p: p-value of the Wald test, n: absolute number of isolates, %: percentage of isolates.
